# Expert consensus on the prevention and treatment of radiochemotherapy-induced oral mucositis

**DOI:** 10.1038/s41368-025-00382-8

**Published:** 2025-07-15

**Authors:** Juan Xia, Xiaoan Tao, Qinchao Hu, Wei Luo, Xiuzhen Tong, Gang Zhou, Hongmei Zhou, Hong Hua, Guoyao Tang, Tong Wu, Qianming Chen, Yuan Fan, Xiaobing Guan, Hongwei Liu, Chaosu Hu, Yongmei Zhou, Xuemin Shen, Lan Wu, Xin Zeng, Qing Liu, Renchuan Tao, Yuan He, Yang Cai, Wenmei Wang, Ying Zhang, Yingfang Wu, Minhai Nie, Xin Jin, Xiufeng Wei, Yongzhan Nie, Changqing Yuan, Bin Cheng

**Affiliations:** 1 Department of Oral Medicine, Hospital of Stomatology, Guanghua School of Stomatology, Sun Yat-Sen University, Guangdong Provincial Key Laboratory of Stomatology, Guangzhou, China; 2 Department of Radiation Oncology, State Key Laboratory of Oncology in South China, Collaborative Innovation Center for Cancer Medicine, Sun Yat-sen University Cancer Center, Guangzhou, China; 3 Department of Hematology, The First Affiliated Hospital, Sun Yat-sen University, Guangzhou, China; 4 Department of Oral Medicine, School and Hospital of Stomatology, Wuhan University, Wuhan, China; 5 State Key Laboratory of Oral Diseases, National Clinical Research Center for Oral Diseases, Department of Oral Medicine, West China Hospital of Stomatology, Sichuan University, Chengdu, China; 6 Department of Oral Medicine, Peking University School and Hospital of Stomatology, Beijing, China; 7 Department of Stomatology, Xinhua Hospital, Shanghai Jiao Tong University School of Medicine, Shanghai, China; 8 Stomatology Hospital, School of Stomatology, Zhejiang University School of Medicine, Zhejiang Provincial Clinical Research Center for Oral Diseases, Key Laboratory of Oral Biomedical Research of Zhejiang Province, Cancer Center of Zhejiang University, Hangzhou, China; 9 Department of Oral Medicine, The Affiliated Stomatological Hospital of Nanjing Medical University, Nanjing, China; 10 Beijing Stomatological Hospital, Capital Medical University, Beijing, China; 11 Department of Radiation Oncology, Fudan University Shanghai Cancer Centre, Department of Oncology, Shanghai Medical College, Fudan University, Shanghai, China; 12 Department of Oral Mucosal Diseases, Shanghai Ninth People’s Hospital, College of Stomatology, Shanghai Jiao Tong University School of Medicine, Shanghai, China; 13 The Third Affiliated Hospital of Air Force Medical University, Xi’an, China; 14 Department of Periodontics and Oral Medicine, College of Stomatology, Guangxi Medical University, Nanning, China; 15 Shanghai Engineering Research Center of Tooth Restoration and Regeneration, Department of Oral Medicine, School of Stomatology, Tongji University, Shanghai, China; 16 Hospital and School of Stomatology, Guizhou Medical University, Guiyang, China; 17 Department of Oral Medicine, Nanjing Stomatological Hospital, Medical School of Nanjing University, Nanjing, China; 18 Department of Oral Emergency and Mucosal Diseases, School and Hospital of Stomatology, China Medical University, Shenyang, China; 19 Centre of Stomatology, Xiangya Hospital, Cental South University, Changsha, China; 20 Department of Periodontics & Oral Mucosal Diseases, The Affiliated Stomatology Hospital of Southwest Medical University, Luzhou, China; 21 Chongqing Key Laboratory of Oral Diseases and Biomedical Sciences, College of Stomatology, Chongqing Medical University, Chongqing, China; 22 School of Stomatology, Jilin University, Changchun, China; 23 State Key Laboratory of Cancer Biology, Xijing Hospital, Fourth Military Medical University, Xi’an, China; 24 Department of Stomatology, The Affiliated Hospital of Qingdao University, Qingdao University, Qingdao, China

**Keywords:** Health care, Mucositis, Oral manifestations, Cancer

## Abstract

Radiochemotherapy-induced oral mucositis (OM) is a common oral complication in patients with tumors following head and neck radiotherapy or chemotherapy. Erosion and ulcers are the main features of OM that seriously affect the quality of life of patients and even the progress of tumor treatment. To date, differences in clinical prevention and treatment plans for OM have been noted among doctors of various specialties, which has increased the uncertainty of treatment effects. On the basis of current research evidence, this expert consensus outlines risk factors, clinical manifestations, clinical grading, ancillary examinations, diagnostic basis, prevention and treatment strategies and efficacy indicators for OM. In addition to strategies such as basic oral care, anti-inflammatory and analgesic agents, anti-infective agents, pro-healing agents, and photobiotherapy recommended in previous guidelines, we also emphasize the role of traditional Chinese medicine in OM prevention and treatment. This expert consensus aims to provide references and guidance for dental physicians and oncologists in formulating strategies for OM prevention, diagnosis, and treatment, standardizing clinical practice, reducing OM occurrence, promoting healing, and improving the quality of life of patients.

## Introduction

Radiochemotherapy-induced oral mucositis (OM) refers to mucosal lesions characterized by congestion, erosion, ulcers and atrophy that occur in the oral cavity of patients treated with head and neck radiotherapy (RT), chemotherapy (CT), or targeted drugs. Epidemiological data show that the incidence of OM in patients undergoing radiochemotherapy for head and neck tumors ranges from 59.4% to 100%.^1^ The incidence of OM varies considerably in patients undergoing CT and is related to the type and dose of chemotherapeutic agents. For example, the incidence of OM in patients undergoing high-dose CT prior to hematopoietic stem cell transplantation (HSCT) can be 70% or more.^1^ OM can lead to pain, difficulty eating, dry mouth, and taste disorders, which seriously affect the survival of patients and even force the interruption of tumor treatment.^1–3^ Therefore, effective prevention and intervention in OM development are of great clinical importance. In recent years, oral mucositis caused by molecular targeted therapy, immunotherapy and other biological therapy strategies has gradually increased, and the clinical treatment principles and significance of OM are the same. Thus, it is also included in tumor therapy-related oral mucositis.

The first evidence-based clinical practice guidelines related to OM were published in 2004 by the Multinational Association of Supportive Care in Cancer and the International Society of Oral Oncology (MASCC/ISOO).^4–7^ Subsequently, relevant guidelines were published by the European Society for Medical Oncology (ESMO) and the Cochrane Collaboration (UK).^8,9^ The China Society for Radiation Oncology (CSTRO) in 2019 and the Antineoplastic Agents Safety Management Committee of the Chinese Society of Clinical Oncology (CSCO) in 2021 took the lead in publishing relevant expert consensuses.^10,11^ However, to date, there has been a lack of in-depth and comprehensive strategies for the prevention, diagnosis, and treatment of this disease from the perspective of oral specialty practices, a sufficient understanding of the important role of oral health status in the prevention and treatment of OM, and guidance for nondental specialists and caregivers. Therefore, based on of evidence-based medicine as well as existing clinical practice guidelines and expert consensus worldwide, this expert consensus is formulated from the point of view of stomatology. It combines experts and scholars from stomatology, oncology, radiology and other disciplines to develop an expert consensus on the clinical diagnosis and treatment of OM that is clinically instructive and consistent with the national conditions of China. Consensus among experts is reached using the Delphi method. This consensus adds new content, including auxiliary examination, diagnosis and differential diagnosis, and efficacy indicators. New interventions, such as topical glucocorticoids,^12,13^ recombinant bovine basic fibroblast growth factor (rb-bFGF),^14–16^ and elemental diets,^17,18^ are recommended in this consensus. Some traditional Chinese medicines have also shown good efficacy in the prevention and treatment of OM, such as Shuanghua Baihe tablets^19,20^ and Kangfuxin liquid^21–23^; we also discuss and recommend these new intervention strategies.

## Epidemiological Characteristics And Pathogenesis

### Risk factors

**Fig. 1:**
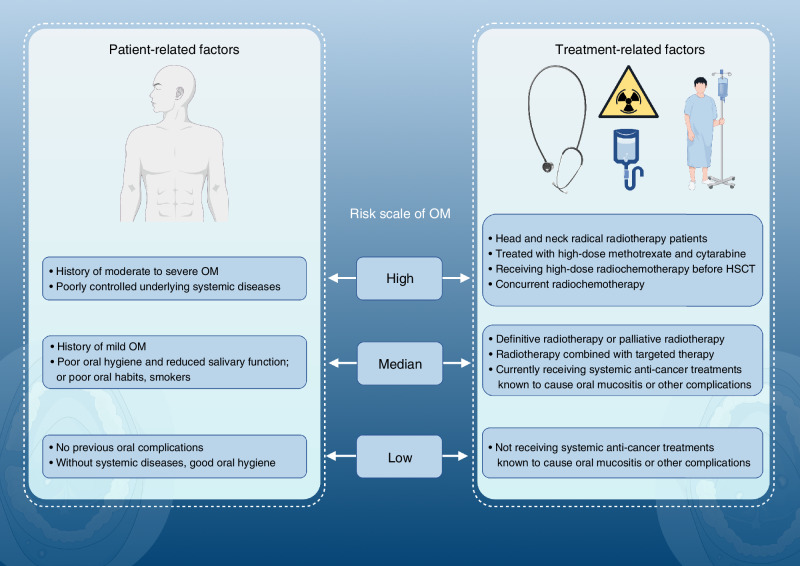
Risk level assessment of radiochemotherapy-induced oral mucositis. Created using Figdraw

The risk factors for OM can be categorized into patient-related factors and treatment-related factors (Fig. 1).

#### Patient-related factors

These factors include previous onset of OM after oncologic treatment, poor oral hygiene, decreased salivary secretion function, poor oral habits, smoking, and poorly controlled underlying systemic disease. The correlation between patient age/sex and the occurrence of OM is controversial.^1^

#### Treatment-related factors

**RT** mainly includes the following: transillumination site, irradiation dose, irradiation volume, fractionated RT and radiation types.^10,11,24–34^ For example, more than 90% of patients treated with systemic RT experience OM, and at least 33% of patients undergoing local RT for head and neck cancer (HNC) developed oral mucositis.^27–29^ In addition, the mode of radiation also affects OM occurrence. The lowest incidence of OM is 16.9% in patients treated with intensity-modulated radiotherapy (IMRT).^30–32^ The incidence of OM with proton beam radiotherapy (PBRT) is comparable to that of conventional RT for the head and neck region, which is 33.4%.^32^ However, the incidence of OM in patients undergoing hyperfractionated RT and accelerated fractionation treatment remains high, at approximately 94% and 71.4%–93.1%, respectively.^28,29,33^ It is generally believed that when the oral cavity is exposed to high doses of RT ((58.8 ± 2.2) Gy), the incidence of severe mucositis of grade 3 or above (mucosal patchy ulcers with significant bleeding and severe pain, affecting eating, and even facing life-threatening risks requiring urgent treatment) can reach 45.8%.^34^

CT mainly includes categories, dosages, regimens and other factors;^11,24^ concurrent RT; high-dose CT or RT administered prior to HSCT; and targeted therapy drugs in combination with radiochemotherapy.^11,35^ The incidence of OM in individuals receiving traditional TAC (docetaxel, adriamycin, cyclophosphamide) regimens can reach 60%, whereas dose-intensive regimens reduce the incidence to 30%.^24^ The incidence of severe mucositis at grade 3 or higher in chemoradiotherapy regimens is as high as 45%; however, this can be reduced to 26% by using IMRT.^36^ The incidence of OM varies when targeted therapy is employed. Specifically, mammalian target of rapamycin (mTOR) and human epidermal growth factor receptor (HER) inhibitors, such as everolimus and afatinib, are associated with occurrence rates ranging from 24%–64% and 25%–72.1%, respectively.^35,37,38^ The morbidity rate of epidermal growth factor receptor (EGFR) inhibitors varies significantly, with dacomitinib reaching values of 37%–41%.^39,40^ Cetuximab potentially reaches a maximum of 37%,^41^ whereas erlotinib has the lowest morbidity, which is typically controlled within 1%.^42^ In comparison, angiogenesis inhibitors, anaplastic lymphoma kinase (ALK) inhibitors, and tyrosine kinase inhibitors all maintain lower OM rates of approximately 29% and rates of severe mucositis of grade 3 or higher of 4%.^43–45^ The incidence of OM with bevacizumab is only 4%, and there have been no reported cases of severe mucositis of grade 3 or higher.^46^

Considering patients’ own factors and treatment-related factors, clinicians can assess the risk of developing OM before tumor treatment based on 3 risk classes: high, medium and low (Fig. 1). Depending on different risk scales, various prevention and treatment strategies can be employed.

### Pathology and pathogenesis

OM can be divided into acute and chronic forms. The acute phase manifests as tissue edema, capillary dilatation, necrosis and rupture of epithelial cells, and exudation of fibrin and blood cells. Sonis et al. summarized the developmental pathological process of mucositis based on the following five stages: the initiation stage, initial damage response stage, signal amplification stage, ulceration stage, and repair stage.^47^

#### Initiation stage

Mucosal tissues quickly enter the initial phase after exposure to RT or CT. At this point, DNA damage is commonly observed in epithelial basal cells and submucosal cells, and DNA double-strand breaks are noted in endothelial cells.^48^ Non-DNA damage occurs simultaneously and often manifests as a dramatic increase in the levels of reactive oxygen species (ROS) associated with cellular damage, leading to oxidative stress within cells.^48,49^ Lymphocyte activation is mediated by the innate immune response of an organism. The initiation phase is the basis for mucositis development, and effective interventions targeting this phase can prevent further mucosal tissue damage.

#### Initial damage response stage

Elevated ROS levels activate transcription factors such as P53, Wnt and NF-κB^50,51^ and induce changes in the expression of inflammatory mediators, such as tumor necrosis factor (TNF-α), interleukins (ILs), cyclo-oxygenase 2 (COX-2), and cell adhesion molecule (CAMs).^52,53^ In addition, c-Jun and c-Jun N-terminal kinase (JNK) expression is upregulated,^54^ activating other transcription factors, such as nuclear factor erythroid 2-related factor 2 (NRF2). The accumulation of TNF-α, IL-6 and IL-1β in the mucosal epithelium and connective tissues leads to increased oxidative stress, basal cell death, and tissue destruction. The Toll-like receptor (TLR) signaling pathway and various kinase pathways, such as the mitogen-activated protein kinase (MAPK) pathway, play a role in this process.^55^ Broken DNA strands caused by radiochemotherapy can directly activate the intracellular ceramide pathway, leading to apoptosis as well as increased permeability of cell membranes.^56^ Moreover, elevated ROS levels activate sphingomyelinase or ceramide synthetase, which hydrolyses the cell membrane lipid sphingomyelin, leading to increased intracellular ceramide levels. Fibroblasts secrete matrix metalloproteinase (MMP) family proteins, such as MMP1 and MMP3, which disrupt the epithelial basement membrane, hydrolyze the subepithelial collagenous matrix, and promote the spread of inflammatory factors to deeper layers of mucosal tissue.

#### Signal amplification stage

The persistent damage to mucosal tissue caused by radiochemotherapy manifests as positive feedback regulation and creates a vicious cycle of inflammatory factors affecting proinflammatory signaling pathways. This prompts entry into the signal amplification phase of OM. For example, exposure to cytotoxic rays or drugs leads to NF-κB signaling pathway activation. The expression of the proinflammatory cytokines TNF-α and IL-1β increases, and these factors are released extracellularly, triggering NF-κB pathway activation in adjacent cells. This results in the upregulation of MAPK, COX2, and tyrosine kinase expression. TNF-α also contributes to the upregulation of ceramides through sphingomyelinase activation, leading to cell apoptosis. Postapoptotic debris and pathogenic microorganisms infiltrating loose intercellular adhesion channels activate the immune system and further activate the NF-κB pathway.^57,58^ Patients at this pathological stage have not yet exhibited significant clinical signs; however, in severe cases, mucosal erythema may be observed.

#### Ulceration stage

Continuous secretion of inflammatory factors leads to severe mucosal tissue damage, loss of epithelial integrity, and ulcer formation. Owing to the presence of exposed nerve endings, patients experience significant pain, which results in a decreased ability for self-oral hygiene management. Many pathogenic microorganisms colonize the ulcer surface, and their toxic factors, such as cell wall components, penetrate into deeper submucosal tissues through the ulcer surface. This prompts lymphocytes, macrophages, and neutrophils to infiltrate and aggregate in this area, where they continuously secrete large amounts of proinflammatory and proapoptotic factors, leading to more extensive cell apoptosis and tissue disintegration.^59^ In severe cases, it can lead to systemic infection.

#### Repair stage

At the end of radiochemotherapy, damaged mucosal tissue has the potential to promote wound healing and self-repair. Epithelial cell proliferation and migration led by the submucosal matrix and mesenchymal-related signals repair mucosal epithelium continuity, maintain redox balance, and remodel the oral microbial ecology. This process is related to the regimen, use of concurrent CT, dosage and duration of radiochemotherapy. However, the genome of the newly produced mucosal epithelium differs from that of the original mucosal epithelium, perhaps resulting in a lower tissue tolerance threshold to radiochemotherapy.

## Clinical Manifestations

**Fig. 2:**
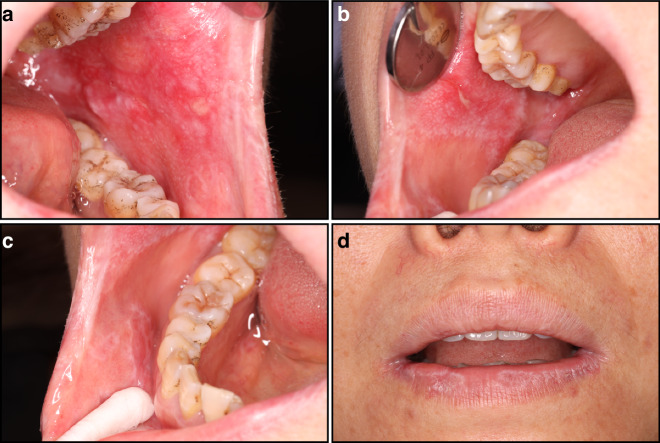
Radiotherapy-induced oral mucositis. Female, 3 months after surgery and radiotherapy for the breast tumor. Painful oral erosions appeared during radiotherapy and persisted for a long time, accompanied by dry mouth. Large areas of congestion and scattered erosions were observed in the cheeks, with fine white lines around the cheeks. White lines with a few scales were observed on the lips. **a** Left cheek; **b** upper right cheek; **c** lower right cheek; **d** lips

When patients with radiotherapy-induced oral mucositis (RIOM) receive a cumulative dose of 10 Gy, they often experience a burning sensation and eating irritation. When the cumulative dose reaches 30 Gy, they may suffer from severe pain, xerostomia, halitosis, and difficulty eating. In severe cases, they may endure dysgeusia and restricted opening of the mouth. A few patients may experience alopecia, pigmentation and fibrosis in the irradiated field. In the acute stage, congestion, edema, erosion, ulceration, and pseudomembrane formation may occur in the oral mucosa of patients with RIOM, and the lesions gradually subside and heal when RT ends (Fig. 2). In severe cases, these signs and symptoms may last up to 2 or 5–7 weeks after RT ends.^1^ Chronic RIOM may also develop up to two years after the completion of radiotherapy and is characterized by secondary damage resulting from extensive atrophy of the salivary glands. Primary clinical manifestations include xerostomia and dysgeusia, with cardinal physical signs presenting as widespread atrophy, thinning, and congestion of the oral mucosa. Chronic RIOM is frequently complicated by concurrent *Candida* infections. Patients typically exhibit dermatological alterations in irradiated skin areas, including dryness, desquamation, hyperpigmentation, and subcutaneous fibrosis.

**Fig. 3:**
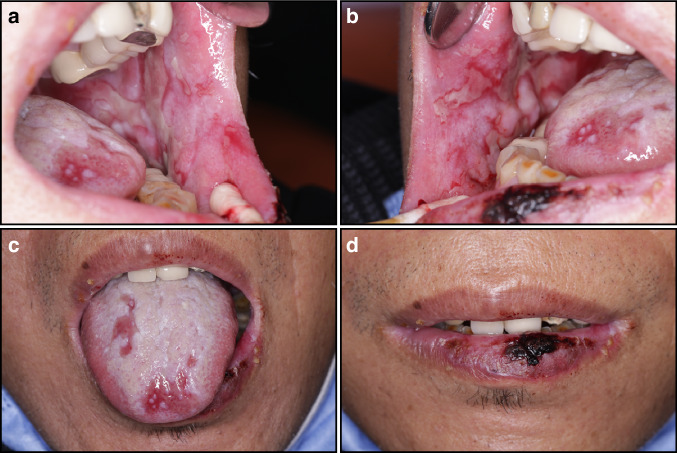
Chemotherapy-induced oral mucositis. Male, postoperative liver cancer and were taking chemotherapy drugs for 9 months, large erosions and ulcers were observed on both the cheeks and the back of the tongue, and thicker blood crusts were observed on the lower lip. **a** Left cheek; **b** right cheek; **c** dorsum of the tongue; **d** lips

Chemotherapy-induced oral mucositis (CIOM) usually begins with a sense of local burning or stinging in the mucosa approximately 4 to 5 days after the initiation of CT, with pain symptoms worsening after 7 to 10 days and culminating approximately 2 weeks later.^11^ The signs are similar to those in the acute phase of RIOM, which mainly manifests as erosion, ulceration, pseudomembranes, or crust (Fig. 3). If the lesion is not infected, it normally resolves within three weeks following treatment. When accompanied by a secondary infection, the oral mucosa shows clinical symptoms of diseases such as *Candida albicans* infection and herpes simplex virus infection.

The clinical manifestations of OM induced by molecular targeted drugs are related to the drug category. For example, mucosal ulceration occurs 10 days after mTOR inhibitor dosing but generally resolves within 1 week.^35,60^ EGFR inhibitors are associated with the development of ulcers 2–3 weeks after dosing, and some patients may experience pain, dysphagia, and dysgeusia.^35,61^ Vascular endothelial growth factor (VEGF) inhibitors cause diffuse oral mucosal sensory abnormalities, mucosal congestion, or ulceration within the first week of dosing.^62,63^ In the context of PD-1/PD-L1 immune checkpoint inhibitors such as navulizumab, OM typically occurs 3 months after the initiation of treatment and is characterized by a mossy lesion with symmetrically distributed white reticulation, which may be accompanied by mild erythema and/or ulceration.^64–66^

**Fig. 4:**
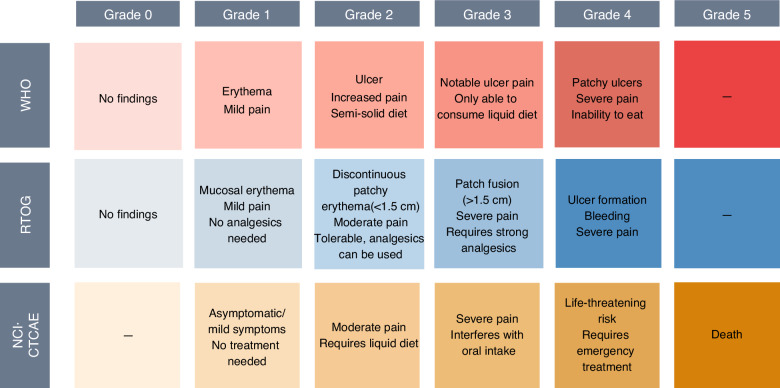
Criteria and Illustrations for the Clinical Grade of Oral Mucositis. WHO World Health Organization, RTOG Radiation Therapy Oncology Group, NCI National Cancer Institute, CTCAE Common Terminology Criteria for Adverse Events

OM is graded according to the signs and symptoms of patients. Currently, the commonly used grading criteria for OM include the World Health Organization (WHO) Oral Toxicity Scale, the Radiation Therapy Oncology Group (RTOG) scoring criteria for acute RIOM, and the National Cancer Institute’s Common Terminology Criteria for Adverse Events (NCI-CTCAE) **(**Fig. 4). The WHO grading standard focuses more on the assessment of eating conditions, whereas the RTOG and NCI-CTCAE criteria emphasize the evaluation of oral pathological and physiological conditions. RTOG classifies toxicity into acute (within 1–3 months) and late (after 3 months) phases, which are generally used for patients undergoing RT alone. NCI-CTCAE is generally used for patients undergoing comprehensive treatments such as concurrent chemoradiotherapy. Although only the WHO and RTOG criteria have been formally adopted through expert voting, the NCI-CTCAE criteria remain included in this consensus according to the recommendations of oncology and radiology experts owing to their critical importance in clinical application. These three criteria are collectively presented to serve as a comprehensive reference framework for guiding clinical practice and research implementation. To date, no evidence is available to suggest which grading criteria are superior. Multiple grading standards are recommended for comprehensive assessment.

## Diagnosis and Differential Diagnosis

The diagnostic criteria for OM include a history of radiochemotherapy, a history of targeted drug therapy or radiation exposure, an association between the onset timing of oral mucosal damage and radiochemotherapy regimens, and typical clinical signs and symptoms of oral mucosal damage. Differential diagnosis is required mainly for acute episodes of erosive ulcerative lesions, especially those that may appear widely distributed, such as herpetiform recurrent aphthous ulcers, primary herpetic gingivostomatitis, allergic stomatitis, erythema multiforme, oral lichen planus (erosive type), and coccigenic stomatitis. The early erythematous phase of OM should be differentiated from oral candidiasis.^1,67,68^

Notably, patients receiving RT often experience a decline in immune function. When OM presents with erosions, ulcers, and other lesions, it is often associated with microbial infections. If necessary, a complete blood count and cultures for bacteria, fungi, and other pathogens can be performed to identify the type of infection and administer targeted medication, thereby improving clinical efficacy.^1,11^ However, owing to differences in economic levels and medical technical levels among institutions in different regions, performing bacterial/fungal cultures and antibiotic susceptibility tests as necessary prerequisites for the use of antimicrobial drugs to treat OM would significantly increase the cost of medical treatment and the complexity of clinical diagnosis and treatment for patients. Therefore, for those who experience poor anti-infective effects according to empirical routines, antibiotic susceptibility tests should be performed in a timely manner to guide the clinical use of drugs. For example, in the case of IMRT for nasopharyngeal carcinoma, in which the incidence of RIOM is 100% and accompanied infections are common, performing a throat swab culture for bacteria can guide the selection of antibiotics, and the infection control effect is superior to that of empirical medication.^69^ The necessity of viral testing is controversial, and it is not the first choice for auxiliary diagnostic methods.

Several emerging technologies, including molecular diagnostics and artificial intelligence, play significant roles in the diagnosis and prediction of OM. For example, various inflammatory biomarkers, such as TNF-α, IL-1β, and IL-6, are significantly elevated in the saliva of patients undergoing chemoradiotherapy and are positively correlated with OM severity. These biomarkers can serve as auxiliary diagnostic tools or facilitate early identification of high-risk patients.^70^ Furthermore, machine learning methods have demonstrated effectiveness in predicting OM occurrence among patients receiving radiotherapy,^71,72^ chemotherapy^73^ and hematopoietic stem cell transplantation.^74^

## Disease Management Measures

### Disease prevention plan

Effective prevention measures can reduce the incidence and severity of OM, delay lesion onset, alleviate pain, and significantly improve patients’ quality of life. Clinicians can assess the risk level of patients about to undergo radiochemotherapy on the basis of the OM risk assessment (Fig. 1), categorize patients into high-risk, medium-risk, and low-risk groups, and then formulate and implement different preventive measures accordingly.

#### Oral health promotion and management

Patients who were well versed in oral health care and understood the adverse effects of RT and CT had a lower incidence, severity, and duration of OM following their initial treatment. This finding could be attributed to patients’ conscious efforts to keep their oral mucosa moist, maintain oral health and hygiene, and effectively control their oral flora.^75,76^ When OM progresses to a severe stage, patients may encounter a range of biopsychosocial issues, including difficulty eating, nutritional imbalances, social barriers, and painful discomfort, all of which contribute to a loss in an individual’s quality of life. Good oral hygiene education and patient management can significantly reduce the adverse effects and complications of OM, such as reduced salivation, dysphagia, and dysgeusia, and improve quality of life scores (QoLSs).^77^ As OM causes symptoms such as pain, bleeding and limited mouth opening, it can lead to a reduction in patients’ ability to take care of their own oral health. Oral health education enables patients to be aware of the importance of oral health and hygiene care in relieving OM symptoms and improving patients’ self-oral management ability and adherence.^78^ The content of oral health education may include nutritional advice for preventing OM, suggestions for preventing and improving dry mouth, advice on issues such as taste and smell changes, techniques for using toothbrushes and dental floss, and daily self-assessment of their oral mucosa.

Patients who actively and continuously manage their oral health before, during, and after radiochemotherapy and control oral pathogen infections can effectively delay the onset of OM and prevent OM progression to severe stages. Kartin et al. proposed comprehensive self-oral care measures and nutritional plans for patients undergoing radiochemotherapy, which can effectively reduce the severity of OM pain, improve patient malnutrition, and enhance their QoLS.^79^ Brushing teeth, rinsing the mouth, using dental floss and interdental brushes, keeping the oral mucosa moist, and maintaining a low temperature are all measures used to strengthen self-oral management.^80^

For patients who are about to undergo radiochemotherapy, the timing of implementing self-oral management is equally important. Basic oral management measures, including tooth brushing and water rinsing, should begin one week before the start of radiochemotherapy. This can effectively delay OM onset in nasopharyngeal cancer patients undergoing radiochemotherapy, reduce its severity, and result in higher oral health assessment scores. Implementing oral health management measures only the day before starting radiochemotherapy is unlikely to have a significant effect on alleviating oral mucositis.^81^ The frequency of daily oral care (such as the number of rinses) and the selection of products/medications for patients should be adjusted to the various OM grading stages.^75,79^

Before patients undergo radiochemotherapy, a comprehensive and professional oral health examination and assessment by dentists to preemptively identify and eliminate potential risk factors for OM could facilitate the smooth progression of radiochemotherapy and effectively control hematogenous infections.^82^ OM risk can be effectively reduced by weekly professional oral examinations and treatments, including scaling and brushing, nutritional intake counseling, and lifestyle guidance, in breast cancer patients undergoing CT.^83^ A randomized controlled phase III trial revealed that regular professional oral care, such as teeth cleaning, reduces the incidence and severity of OM in breast cancer patients treated with everolimus and exemestane while also improving patients’ tolerance and compliance in managing CT-induced complications.^84^ Professional oral assessment and treatment should include the following six aspects:^85,86^ (1) comprehensive assessment of OM susceptibility factors (oral hygiene, caries, metal restorative materials, and dentures); (2) controlling the susceptibility factors that can cause mucosal or periodontal infections and prevent the development of localized infections and systemic spread of infection (periodontal scaling, removal of residual roots/crowns/affected teeth, conversion of metal to nonmetal fillings, removal of poorly restored restorations, maintenance of oral hygiene); (3) reducing oral discomfort and pain; (4) maintaining basic oral function to support nutritional intake and speech function; (5) managing oral complications from RT and drug-targeted therapies; and (6) enhancing individual quality of life.

#### Topical drug prevention

Despite available evidence that patients receiving professional oral management practices can help prevent OM, approximately 75% of patients still present with adverse signs on the oral mucosa following RT.^84^ For low- and median-risk patients, a combination of oral specialty prophylaxis, such as anti-inflammatory and analgesic agents, healing promotion and coinfection control, is needed.

##### Anti-infective drugs

Chlorhexidine rinses have been shown to play a significant role in antimicrobial therapy for periodontal tissues, as these drugs inhibit bacterial, fungal and viral colonization. As a risk factor for OM, intraoral microbial colonization of pathogenic organisms in combination with cytotoxic therapy plays a significant role in contributing to OM development. Meta-analysis has shown that local application of chlorhexidine rinses effectively prevent CIOM.^87,88^ However, chlorhexidine does not have significant preventive effects on RIOM. Patients in the CT group receiving 0.12% chlorhexidine gargle solution three times a day at 15 mL/dose presented a decreased incidence, faster remission, and reduced severity of OM, whereas those in the RT group had no significant preventive effect. Intraoral microflora colonization was reduced in both groups.^89^ Two additional randomized controlled trials further validated that local application of chlorhexidine does not effectively prevent RIOM^90^ and may even cause more severe OM, as well as more noticeable adverse effects (taste alternation and tooth staining).^91^ Therefore, the local application of chlorhexidine rinses is only recommended for the prevention or mitigation of CIOM, not RIOM.

Sodium bicarbonate removes detached cellular debris from the surface of the oral mucosa, increases the pH, and inhibits acidophilic bacteria. Sodium bicarbonate mouthwash has prophylactic effects against OM and postpones the onset of CIOM in patients undergoing CT for acute leukemia.^92^ However, it is less effective than oral rinsing with benzydamine in preventing RIOM.^93^ It is recommended that 10 g of sodium bicarbonate powder be dissolved in 1 L of sterile water or physiological saline and used at 15 mL/2 min/4 times/day, starting from the day before RT and continuing for two weeks after treatment is completed.

##### Pro-healing drugs

Granulocyte-macrophage colony-stimulating factor (GM-CSF) promotes the aggregation of lymphocytes and myeloid cells, such as macrophages and Langerhans cells, inducing local tissue inflammatory responses and stimulating macrophages to secrete pro-healing molecules. Additionally, GM-CSF stimulates the growth of keratinocytes and fibroblasts, promoting ulcer surface repair and wound healing.^94^ The use of GM-CSF for oral administration or gargling has a definite effect on preventing RIOM, effectively delaying the progression of RIOM to severe stages.^95–97^ A GM-CSF oral rinse (150 g GM-CSF in 100 mL of 0.9% NaCl solution) was prepared and gargled 4 times a day from the pretreatment date (25 mL per wash >3 min) and swallowed in small amounts after gargling. However, the effectiveness of subcutaneous GM-CSF injections in preventing RIOM is controversial.^98–102^

Topical application of Kangfuxin liquid significantly reduces the incidence and severity of RIOM.^22^ Kangfuxin liquid can increase the function and quantity of neutrophils in wounds while promoting the secretion and deposition of the extracellular matrix, expediting the healing process. Starting from the day of RT, 10 mL Kangfuxin liquid is gargled for 3 minutes, and the mixture is then swallowed slowly, three times a day, until the end of RT. When needed, Kangfuxin liquid ice cubes can be used for buccal administration.^23^

##### Anti-inflammatory and analgesic drugs

As nonsteroidal anti-inflammatory drugs, benzydamine inhibits the secretion of TNF-α and IL-1 from local mucosal tissues, acting as anti-inflammatory, antibacterial and local analgesic agents.^103,104^ For head and neck squamous cell carcinoma (HNSCC) patients receiving moderate doses of RT (a total radiation dose of up to 50 Gy, with a daily radiation dose less than 2.2 Gy), the use of benzydamine oral rinses could reduce the incidence of erythema and ulcers by approximately 30%, relieve patients’ pain, and prevent RIOM effectively and safely.^105,106^ For patients receiving concurrent chemoradiotherapy, a benzydamine oral rinse is also useful for preventing OM and controlling oral mucosal infections.^93,107,108^ The recommended usage is as follows. For patients receiving moderate radiation doses (<50 Gy) or CT, a 0.15% benzydamine oral rinse (15 mL/2 min/dose) should be used 4-8 times per day starting on the day of treatment. The oral rinse can be diluted proportionally if the solution is too thick.

Rebamipide inhibits the production of reactive oxygen species by inflammatory cells and scavenges hydroxyl radicals.^109,110^ The use of oral rinses with rebamipide may prolong the onset and reduce the severity of OM in RT patients.^111^ The recommended concentration for patients receiving radiation for HNC is 4%.^112^ It can be started three days before radiochemotherapy, with a dosage of 5 mL for 30 s per rinse, six times a day. The rinse should be continued for three months.

#### Systemic drug prevention

For high-risk patients with tumors prone to OM, in addition to necessary oral hygiene management and local treatment, systemic pro-healing and anti-inflammatory agents are recommended to prevent the occurrence of moderate or severe OM.

Intravenous keratinocyte growth factor-1 (KGF-1) is effective in preventing OM. However, its outpatient application is limited, and it is mostly used in hospitalized patients. Intravenous injection of KGF-1 can reduce the incidence of moderate to severe OM in patients receiving head and neck radiochemotherapy, prevent the occurrence of OM, and delay the onset of severe OM.^113,114^ For acute leukemia patients who require CT, KGF-1 can also play a role in preventing the exacerbation of OM.^115^ Currently, a consensus on the dosage and frequency of KGF-1 usage is not available. However, a common dose used in clinical trials is 360 μg/kg, and the number of intravenous injections is often six. It can be administered once daily from 3 days before to 3 days after radiochemotherapy initiation or once a week starting one week before radiochemotherapy and continuing weekly thereafter.^9^

Glutamine can alleviate the oxidative damage caused by ionizing radiation by inhibiting the secretion of proinflammatory factors. It activates collagen synthesis, provides the necessary energy for cell proliferation, and promotes mucosal wound healing.^116^ For patients with HNC undergoing RT, oral glutamine (10 g/1 000 mL, taken 2 h before RT) can effectively prevent and remedy RIOM, significantly alleviate the severity of OM, reduce opioid analgesic use and decrease the likelihood of nasogastric tube feeding and treatment interruptions.^117,118^ In patients with solid tumors during CT, treatment with an oral glutamine suspension has also been shown to be effective in relieving moderate to severe OM and reducing patient pain (2 g/M^2^, twice a day).^119,120^

Pilocarpine has effects similar to those of acetylcholine, stimulating the secretion of saliva and sweat and preventing xerostomia caused by RIOM. Studies have shown that oral administration of pilocarpine significantly reduces the incidence of severe RIOM (grade ≥3) in HNC patients.^13,121^ However, pilocarpine does not have significant effects on patients with oral squamous cell carcinoma receiving concurrent chemoradiotherapy.^13^ Pilocarpine can significantly reduce the severity of OM in patients with solid tumors and acute leukemia undergoing chemotherapy,^122^ but it does not provide benefits for patients undergoing pretransplant conditioning for HSCT.^123^ It can be administered orally at 5 mg/dose 3 times/day prior to treatment and reduced to 2.5 mg/dose 4 times/day if the patient sweats significantly.

As traditional Chinese medicines, Shuanghua Baihe tablets contain berberine as a Coptis Rhizoma component and have been proven to have antibacterial, antifungal, antioxidant, and anti-inflammatory properties. For patients undergoing RT in the head and neck region, Shuanghua Baihe tablets can be used to reduce the incidence of OM, alleviate the degree of pain, and shorten the healing time.^19,20^ The tablets can be taken orally from the first day of treatment (4 tablets/dose, 3 times/day).

#### Photobiotherapy prevention

Low-level laser therapy (LLLT) can directly act on cellular mitochondria, maintain cellular homeostasis, promote cell proliferation and collagen synthesis, increase cellular metabolism, accelerate wound healing and tissue repair, induce angiogenesis, enhance leucocyte activity, reduce damage caused by radiochemotherapy to tissues, and have analgesic effects.^124^ For pediatric patients undergoing HSCT, high-dose radiochemotherapy or radiochemotherapy for head and neck tumors, a low-level laser (620–750 nm) is beneficial for preventing and controlling severe OM (grade ≥3).^125–127^ For patients with head and neck tumors undergoing chemoradiotherapy, LLLT has also been proposed to have a role in preventing OM.^128^ Owing to the lack of well-designed and large-scale clinical trials, the parameters for LLLT mostly depend on the experience of clinical physicians. For patients with HNC undergoing chemoradiotherapy, the parameters for preventing OM in multiple clinical trials of LLLT include a wavelength ranging from 632.8 to 660 nm, an energy density ranging from 2.5 to 4 J/cm^2^, and power ranging from 10 to 100 mW.^129^ Notably, individual studies reported that LTTT was not effective in preventing radiochemotherapy-induced OM,^130^ but most studies support its effectiveness. In addition, some experimental studies suggest that photobiomodulation has a carcinogenic effect,^131–133^ but current clinical evidence shows that photobiomodulation has good safety.^134,135^ Nonetheless, clinicians should inform patients of the potential risks of photobiomodulation therapy before its use.

Additionally, the use of cryotherapy^136^ and standard oral care^137,138^ in conjunction with LLLT are advisable for OM prevention.

#### Others

Saline solution can serve as a mild mouthwash, helping patients maintain oral health and hygiene, as well as improving their comfort level.^139^ It has been suggested that topical application of sucralfate mouthwash or oral administration of sucralfate in patients can be effective in preventing OM;^140–143^ however, some studies report the opposite conclusion.^144,145^ Cryotherapy refers to chewing ice chips before and after CT to reduce the temperature of the oral mucosa. This can constrict mucosal blood vessels and decrease blood flow, thereby reducing the effects of CT drugs on the oral mucosa and preventing OM occurrence.^146–150^ However, although cryotherapy has the advantages of low cost and ease of implementation, its preventative efficacy is limited by the drug’s half-life, and it cannot replace the important role of patient oral care education and monitoring in the prevention of OM. Additionally, patients with nasopharyngeal carcinoma who undergo RT can wear personalized oral stents to reduce the radiation dose received by the oral mucosa; this can help to preserve healthy oral mucosa and potentially avoid RIOM.^151^

### Disease control plan

#### Oral health management

On the basis of sufficient evidence available,^83,84,152,153^ professional oral management by dental specialists during RT for tumors is conducive to monitoring the health condition of the oral mucosa, relieving symptoms and promoting healing. Specific measures include (1) conducting a comprehensive oral examination weekly during the treatment period to closely monitor oral conditions, with particular attention given to the second week after CT, which is a high-incidence period for OM; (2) taking appropriate management measures based on oral assessment, such as periodontal scaling, crown surface polishing, tongue microbiome control, and the use of antimicrobial mouthwashes or corticosteroid ointments; and (3) instructing and checking the patient’s tooth brushing method by a doctor or nurse, advising patients to brush their teeth gently once in the morning and once before bed, brushing gently and avoiding abrasing soft tissues. In addition, if patients have a tendency for severe gum or mucosal bleeding, using cotton swabs to remove deposits from the surface of teeth and mucosa.

For special populations such as pediatric patients, owing to their weaker ability to control their own oral hygiene, it is more important to emphasize the role of guardians in helping children achieve self-oral hygiene management. Additionally, to closely monitor any oral complications that arise in children receiving CT/RT and facilitate antitumor treatment, a variety of oral management strategies should be used.^154,155^ Specific measures include (1) long-term professional oral management by dental specialists; (2) oral health education for patients and their guardians; (3) daily assessment of oral hygiene in hospitalized patients; and (4) the use of various oral management measures, such as compound mouthwashes (antimicrobial + analgesic + glucocorticoid + vitamin B + fluoride-containing), in conjunction with photobiomodulation therapy.

#### Medication

##### Anti-inflammatory drugs, analgesics and antidepressants

Burning, irritation, and pain are common clinical symptoms in patients with OM, resulting in eating, swallowing, and speech disorders, as well as a lower quality of life. Topical applications of doxepin, lidocaine, and tetracaine can effectively alleviate pain symptoms associated with OM.

Doxepin is a tricyclic antidepressant that works by blocking sodium ion channels^156^ and preventing stimulus conduction in cutaneous and mucosal nociceptors. It has sedative and analgesic effects when used topically. Several studies have shown that using doxepin oral rinses can provide rapid analgesia when radiochemotherapy patients experience mucosal pain, and the analgesic effect can last for more than 2 hours.^157–160^ The drug may have side effects such as taste disturbances and drowsiness after use, and patients can use it as a sleep aid. Doxepin oral rinse (5 mg/mL) can be used for 1 min; this should be followed by coughing and spitting it out 3–6 times/day.

Lidocaine can also be used to alleviate OM-related pain, which is characterized by rapid onset. In contrast, the analgesic effect lasts for a shorter duration than doxepin (approximately 30 min to 1 h), which may lead to an increased frequency of drug administration.^157,161^ Patients can rinse with a lidocaine solution and then spit it out or apply viscous lidocaine + 1% cocaine directly to painful mucosal areas.^162^

A clinical trial proposed that the use of 1.5% tetracaine gel can safely and effectively reduce RIOM-related pain in a significant proportion of HNC patients.^163^ However, larger randomized controlled trials are needed to investigate the effects of tetracaine compared with those of other topical analgesics.

Topical glucocorticoids are often applied clinically to relieve RIOM symptoms. It has been reported that topical dexamethasone ointment reduces the incidence of severe OM in patients treated with RT for OSCC; however, no substantial efficacy has been observed in patients treated with concurrent chemoradiotherapy.^13^ Triamcinolone acetonide mucoadhesive films have also been shown to be effective in reducing pain in RIOM patients.^12^

When stimulated by radiochemotherapy, mucosal tissues release large amounts of inflammatory factors, free radicals, and oxidative stress, leading to rapid destruction of epithelial tissue. A oral rinse containing 0.2% vitamin E (antioxidant), 0.1% triamcinolone acetonide (anti-inflammatory agent) and 0.2% hyaluronic acid (topical reductant to reduce stimulation of reactive oxygen on mucosal tissues) can achieve good therapeutic effects on OM.^164^ The usage was gargling 2 mL/min, 3 times/day, and not eating or drinking for 15 min afterward.

Protective oral gel is a bioadhesive material that can adhere to mucosal ulcers, quickly forming a protective barrier that provides wound protection and analgesia. Several clinical trials have demonstrated that protective oral gel can adhere to red, swollen and ulcerated oral mucosa of head and neck CT/HSCT patients and provide analgesic effects for a long period of time (6–8 h), effectively relieving regional pain.^165–167^

##### Antimicrobials

Mucosal tissues of patients undergoing radiochemotherapy are prone to damage and disintegration, providing channels for oral bacteria and fungi to invade deeper tissues. Patients often suffer from pain and other antitumor therapy complications, resulting in poor self-cleaning. This can cause residual food debris in the oral cavity, providing sustenance for pathogenic microorganisms. The cell wall and other toxic substances can cause more severe cellular phagocytosis and tissue inflammatory responses. Using chlorhexidine and sodium bicarbonate oral rinses, miconazole mucoadhesive buccal tablets to control the number and activity of bacteria and fungi in the oral cavity can be effective in relieving OM symptoms.

Studies have shown that 0.12% or 0.2% chlorhexidine mouthwash can reduce the duration and severity of OM in patients undergoing chemotherapy.^89,168^ Five percent sodium bicarbonate mouthwash can effectively treat and alleviate OM severity, accelerate lesion healing, and subsequently improve the QoL of cancer patients during CT.^169,170^ Miconazole has strong inhibitory activity against most strains of *Candida*, especially fluconazole-resistant strains.^171^ Miconazole mucoadhesive buccal tablets have been used for oropharyngeal candidiasis in HIV patients^172^ and in patients with HNC undergoing CT.^173^ It is also an effective alternative to oral amphotericin B suspension in patients undergoing high-dose therapy/autologous stem cell transplantation (HDT/ASCT), reducing complications caused by excessively high levels of antifungal agents in blood.^174^

##### Pro-healing drugs

IL-11 promotes peripheral platelet production, stimulates hematopoietic stem cell proliferation, and facilitates ulcer healing. It regulates macrophage function, promotes the secretion of epidermal growth factor, shortens the cell growth cycle, and accelerates tissue repair. IL-11 oral rinses can effectively reduce OM clinical grade, shorten healing tim,^175,176^ and promote a decrease in serum C-reactive protein levels, alleviating pain.^177^ The use of an IL-11 oral rinse (1.5 mg/100 mL) after brushing and rinsing is recommended, and 10 mL should be gargled for 10 min per wash, 4 times/day.

Recombinant bovine basic fibroblast growth factor (rb-bFGF) is effective in reducing the healing time of RIOM lesions.^16^ When used in combination with Kouyanqing granules^14^ or sodium bicarbonate mouthwash,^15^ it has a good therapeutic effect on RIOM in patients undergoing RT for HNC. Notably, given the cell growth-promoting effects of rb-bFGF, clinicians should consider its advantages and disadvantages and make comprehensive decisions regarding its use in patients with oral malignant tumors receiving radiochemotherapy.

Kangfuxin liquids and Shuanghua Baihe tablets are potentially effective in both the prevention and treatment of OM. In patients receiving RT in the head and neck region, Kangfuxin liquid has been shown to significantly reduce OM duration and severity.^21,22^ A randomized controlled trial with a larger sample size also revealed that oral administration of Shuanghua Baihe tablets effectively relieves OM.^19,20^ The usage is consistent with that previously described.

On the basis of the overwhelming evidence available,^117,119,178,179^ oral glutamine is effective in prevention and treatment of OM. The use of 10 g/dose oral glutamine suspension 3 times/day for patients receiving RT and 2.5 g/5 mL/dose glutamine suspension 3 times/day for patients with solid tumors receiving CT can effectively reduce moderate to severe OM.

#### Diet and nutrition

An elemental diet is a chemically refined meal that contains all the easily digestible and absorbable nutrients needed by the body, including amino acids, carbohydrates, vitamins, inorganic salts, trace minerals, and minimal fats. This diet can effectively aid in the early recovery and symptom relief of OM. Oral administration of an elemental diet (containing 79.3% carbohydrates, 17.6% amino acids, 0.6% soybean oil, 2.0% minerals, and 0.5% vitamins) may promote early recovery from OM damage induced by pretreatment of HSCT patients, thereby reducing hospital stays.^18^ Elemental diets can also effectively improve OM in OSCC patients and increase the radiochemotherapy completion rate.^17^

#### Photobiotherapy

LLLT is not only significantly effective in preventing OM but also widely recognized for its ability to relieve OM symptoms. Despite the evident benefits of LLLT, different parameter settings may lead to varying treatment outcomes and poor reproducibility.^7,180^ A systematic review and meta-analysis^181^ reported that LLLT (wavelength of 632.8–685 nm, energy density of 1.3–6.2 J/cm^2^, and power of 5–100 mW) has good clinical outcomes for HNC patients undergoing radiochemotherapy, reducing patient pain and the risk of developing severe OM. Another meta-analysis suggested that^182^ the most commonly used laser parameters for OM treatment include a wavelength of 660 nm, an energy density of 4 J/cm^2^, a power density of 1 W/cm^2^, an energy of 0.16 J, a power of 40 mW, and a spot size of 0.04 cm^2^.

Currently, most clinical trials of LLLT are conducted using intraoral irradiation. Some scholars have proposed that extraoral LLLT can be equivalent to intraoral transillumination in reducing OM clinical grade and pain levels and accelerating the healing time of mucosal damage.^183^ In addition, extraoral LLLT has the benefits of shortening the treatment period (approximately 4 min), improving the cooperation of pediatric patients,^184^ and demonstrating efficacy in the treatment of mucositis of the lips and esophagus.

#### Others

Topical morphine oral rinse is beneficial in relieving OM pain,^185–187^ but its use has limitations. Moreover, studies have shown that morphine can lead to a reduction in cell migration, which in turn inhibits wound healing.^188,189^

Honey and propolis are considered natural antioxidants that effectively block the production of free radicals and act as anti-inflammatory and pro-healing agents. Some studies have suggested that honey/propolis may reduce the progression of OM to severe OM and have abirritation and weight maintenance effects for patients,^190–192^ whereas some clinical trials have reported that the therapeutic effect of honey/propolis does not differ from that of placebo group.^193–195^

**Fig. 5:**
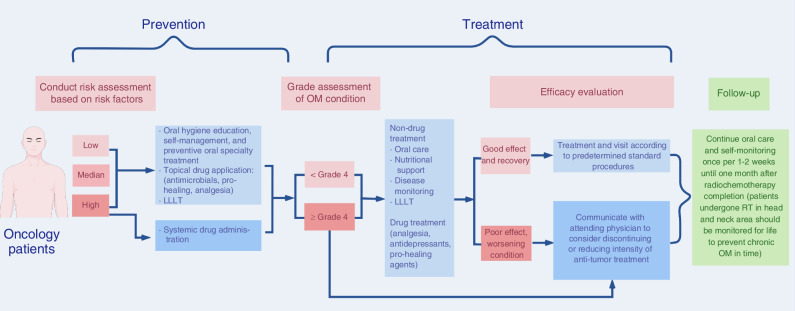
Guidelines for the clinical prevention and management of radiochemotherapy-induced oral mucositis

In summary, clinicians can conduct risk assessments for patients undergoing oncological treatment and choose from the above OM control measures according to the patient’s actual situation based on comprehensive consideration. The clinical guidelines for the prevention and treatment of OM are shown in Fig. 5. If the therapeutic effect is good and recovery is satisfactory, standard treatment and visitation procedures should be followed without the need for additional follow-ups or interventions. If the effect is poor and the patient’s condition worsens, the patient should communicate with an attending physician to consider discontinuing or reducing the intensity of antitumor treatment. After the OM has subsided, the plan should be adjusted to complete subsequent antitumor therapy. Importantly, from the pretreatment phase of radiochemotherapy until one month after anticancer treatment completion, dental specialists should provide continuous oral care for patients and educate patients on self-examination and self-management once every 1–2 weeks. Individuals who have had RT to the head and neck region should be monitored throughout a patient’s life to prevent and treat the occurrence and development of chronic OM over time.

### Indicators of efficacy

The effectiveness of OM clinical preventive and therapeutic measures can be assessed in terms of OM lesion severity and degree of oral pain; additionally, assessments can be supplemented by examining the incidence, duration and safety evaluation of severe oral mucositis (grade 3 or higher).^106,196,197^ The severity of OM lesions can be determined according to clinical grading criteria such as the WHO, RTOG or NCI-CTCAE; the degree of oral pain can be assessed using the visual analog scale (VAS) (0–10), and the pain affect faces scale (PAFS) (0–10). Safety assessments mainly include records of adverse reactions and adverse events.

## Conclusion and Expectation

Oral mucositis is one of the most common and severe complications induced by antitumor therapy and can cause redness, swelling and pain in the oral mucosa of patients, leading to speech and eating difficulties and reducing the quality of life of individuals. In severe cases, it can force interruption of radiochemotherapy plans, interfere with antitumor treatment progression, and extend a patient’s hospital stay. Therefore, clinicians should pay attention to patients with tumors and HSCT who are susceptible to the risk of OM, monitor and manage their oral hygiene and health, provide oral health information and education to them and their families to prevent the occurrence of OM, prevent and control coinfections, alleviate pain, control symptom deterioration and promote the healing process through pharmacological and nonpharmacological means.

This consensus acknowledges several inherent limitations. First, the evidence selection process may carry potential biases due to the possible subjectivity in prioritizing certain research. Second, expert opinions, although valuable, might reflect individual specialty backgrounds or clinical experiences that could influence recommendation alignment. Third, significant knowledge gaps persist in specific clinical areas, potentially limiting the generalizability of recommendations across diverse health care systems. Finally, the incorporation of recommendations involving traditional Chinese medicine into international practice currently faces implementation challenges.

Notably, current clinical research on OM intervention strategies predominantly presents a low evidence hierarchy, underscoring the imperative need for future high-quality randomized controlled trials to establish robust guidance for the clinical management of OM. Furthermore, the implementation of preventive and therapeutic measures should be judiciously tailored to regional economic disparities and variations in health care infrastructure across medical institutions. This adaptive approach ensures optimal patient benefit through context-sensitive application of medical interventions while maintaining rigorous adherence to evidence-based practice standards.
